# The genome of a giant (trevally): *Caranx ignobilis*

**DOI:** 10.46471/gigabyte.67

**Published:** 2022-08-30

**Authors:** Brandon D. Pickett, Jessica R. Glass, Timothy P. Johnson, Perry G. Ridge, John S. K. Kauwe

**Affiliations:** ^1^ Department of Biology, Brigham Young University, Provo, Utah, USA; ^2^ South African Institute for Aquatic Biodiversity, Makhanda, South Africa; ^3^ College of Fisheries and Ocean Sciences, University of Alaska Fairbanks, Fairbanks, Alaska, USA; ^4^ Tim Johnson Gallery, Mesa, Arizona, USA; ^5^ Brigham Young University – Hawai‘i, Laie, Hawai‘i, USA

## Abstract

*Caranx ignobilis*, commonly known as giant kingfish or giant trevally, is a large, reef-associated apex predator. It is a prized sportfish, targeted throughout its tropical and subtropical range in the Indian and Pacific Oceans. It also gained significant interest in aquaculture due to its unusual freshwater tolerance. Here, we present a draft assembly of the estimated 625.92 Mbp nuclear genome of a *C. ignobilis* individual from Hawaiian waters, which host a genetically distinct population. Our 97.4% BUSCO-complete assembly has a contig NG50 of 7.3 Mbp and a scaffold NG50 of 46.3 Mbp. Twenty-five of the 203 scaffolds contain 90% of the genome. We also present noisy, long-read DNA, Hi-C, and RNA-seq datasets, the latter containing eight distinct tissues and can help with annotations and studies of freshwater tolerance. Our genome assembly and its supporting data are valuable tools for ecological and comparative genomics studies of kingfishes and other carangoid fishes.

## Context

The “genomic revolution” continues to rapidly advance our understanding of human evolution and the evolution of non-model organisms [1]. Comparative genomic approaches using whole-genome datasets allow for discoveries at every scale: from genome to chromosome to organism to entire clades of organisms. Genomic datasets of non-model marine teleost fishes (the most diverse clade of vertebrates) are invaluable for investigating evolutionary questions relating to adaptation, selection, genome duplication, and phylogenetic conservatism in vertebrates.

**Figure 1.**
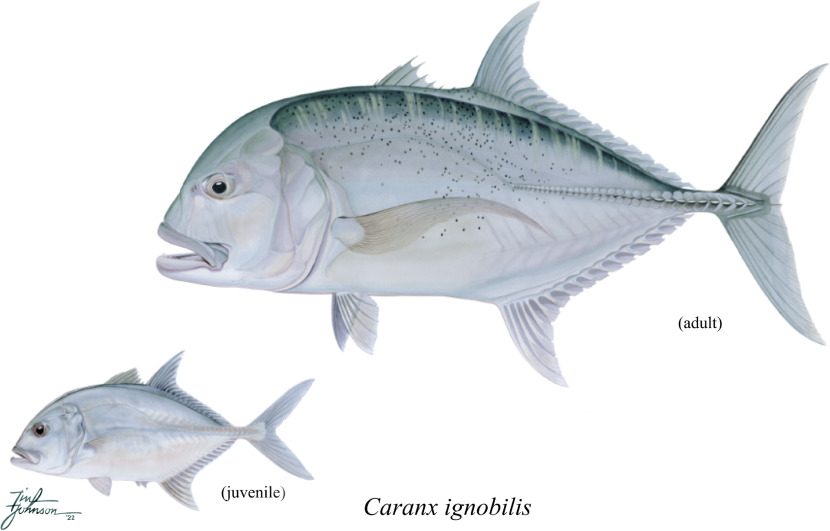
Giant trevally (*Caranx ignobilis*) adult and juvenile. The quantitative morphological data for this illustration of *C. ignobilis* were obtained primarily from Smith-Vaniz (1999) [12]. These were evaluated by the artist who selected the specific values for meristic traits represented in the adult illustration, including the number of lateral-line scutes (32), the number of dorsal-fin rays (20) and spines (9), and the number of anal-fin rays (16) and spines (1*). Each value represents the centermost whole number in the corresponding range reported (within 1). The ratio of body depth to fork length, as depicted (1:2.85), is also at the center of the reported range (1:2.5–3.2). While the literature provides limited physical descriptions (such as the general shape, the color, and the presence of a posterior adipose eyelid), the artist found great benefit in some excellent photographs of live specimens caught and identified by Dr. J. S. K. Kauwe and others. A full-resolution version can be viewed at https://www.timjohnsongallery.com/caranx-ignobilis-illustration (also archived at the Internet Archive (https://web.archive.org) on 30th August 2022). * Smith-Vaniz [12] and others report *C. ignobilis* as having three anal-fin spines (two anterior to and one connected to the lobe of the anal fin), and these are represented in the juvenile illustration. However, the adult is presented here without anterior anal fin spines; they are fully embedded and, therefore, not visible. Although anal-fin-spine embedment (or a corresponding change in spine count) among the adults of *C. ignobilis* is not reported by Smith-Vaniz [12], or indeed by any of the other descriptions we found, it has been reported to often happen to the detached anal-fin spines of Carangids as they grow to adulthood [13]. More importantly, the large majority of the source photos of adult *C. ignobilis* identified and provided by Dr. Kauwe clearly showed the absence of visible, unembedded anterior anal-fin spines. The illustration was rendered accordingly. It may be advisable to update current morphological data sets on *C. ignobilis* to reflect the apparently common phenomenon of anal-fin spine embedment, including the corresponding change in visible spine count. It would also be important to include this information in future published descriptions to prevent confusion or error in identifying adults of the species that would otherwise not match the reported meristic characteristic.

Here, we present a draft genome assembly of a marine teleost, giant trevally (*Caranx ignobilis*; Carangiformes: Carangoidei; Figure 1). This assembly is a valuable resource for the fields of evolutionary biology, ecology, and phylogenetics. *Caranx ignobilis* is a member of the Carangini clade, the most specious subclade within Carangoidei. Carangoid fishes are known for their extreme diversity in morphology and ecology [2, 3]. The giant trevally, specifically, is known to be highly tolerant of freshwater environments. This feature renders this species highly interesting for aquaculture [4–6] and makes it an ideal candidate species to investigate linkages between genotype and phenotype in the context of the freshwater adaptation of marine fishes [7, 8]. *Caranx ignobilis* is an apex predator in tropical and subtropical reefs and coastal environments in the Indian and Pacific Oceans [9], and is heavily targeted by small-scale and recreational fisheries throughout its range. Understanding its evolutionary and ecological role in the ecosystem structure and function is important for fisheries management and the protection of reef and coral ecosystems. Importantly, new putative populations of *C. ignobilis* in the Indian and Pacific Oceans have recently been described using genomic datasets [10]. A highly-continuous genome allows for the inference of demographic history, genomic signals of selection and adaption, and comparative genomic studies with other Carangoid fishes, such as the hybridization with the closely related bluefin trevally, *Caranx melampygus* [11].

 For our *C. ignobilis* assembly, we present the results derived from 58.25 Gbp of Pacific Biosciences (PacBio) single-molecule real-time (SMRT) sequencing data. The Illumina paired-end sequencing data were also generated with libraries for both RNA-seq and Hi-C, totaling 347.6 Gbp. Both datasets were used for scaffolding purposes and are valuable individually. The estimated genome size is 625.92 Mbp [14, 15], of which 96.7% is covered by known bases in the primary haploid assembly. In addition to being highly contiguous, our genome assembly contains complete, unduplicated copies of >95% of the expected single-copy orthologs, suggesting the assembly is reasonably complete. This draft assembly and the supporting sequencing datasets are sufficiently high-quality to serve as valuable resources for a variety of prospective comparative and population genomics studies.

## Methods

An overview of the methods used in this study is provided here. Where appropriate, additional details, such as the code of custom scripts and the commands used to run software tools, are provided in a file in GigaDB [16].

### Sample acquisition and sequencing

Blood, brain, eye, fin, gill, heart, kidney, liver, and muscle tissues from one *C. ignobilis* (NCBI:txid376895; Fishbase ID: 1985) individual were collected off the coast of O‘ahu (near Kaneohe, Hawai‘i, USA) in April 2019. The blood sample was preserved in ethylenediaminetetraacetic acid (EDTA), and the other tissue samples were flash-frozen in liquid nitrogen. All samples were packaged in dry ice for transportation to Brigham Young University (BYU; Provo, Utah, USA) and stored at −80 °C until sequencing. The blood sample was used to create the Omni-C dataset. All the non-blood tissue samples were used for short-read RNA sequencing; the heart tissue was also used for long-read DNA sequencing.

DNA was prepared for long-read sequencing with a Pacific Biosciences (PacBio; Menlo Park, California, USA; https://www.pacb.com) SMRTbell Library kit, adhering to the following protocol: “Procedure & Checklist – Preparing gDNA Libraries Using the SMRTbell Express Template Preparation Kit 2.0” [17]. The continuous long-read (CLR) sequencing was performed on seven SMRT cells for a 10-h movie on the PacBio Sequel at the BYU DNA Sequencing Center (DNASC; https://dnasc.byu.edu), a PacBio Certified Service Provider. The RNA libraries were prepared with Roche (Basel, Switzerland; https://sequencing.roche.com) KAPA Stranded RNA-seq kits, following recommended protocols. The paired-end sequencing was performed in High Output mode for 125 cycles, with the eight samples across two lanes, on an Illumina (San Diego, California, USA; https://www.illumina.com) Hi-Seq 2500 (RRID:SCR_016383) at the DNASC. Finally, the “Omni-C Proximity Ligation Assay Protocol” version 1.0 was followed using a Dovetail Genomics Omni-C kit to prepare the DNA for Illumina Paired-end sequencing. Adapters were obtained from Integrated DNA Technologies, and sequencing proceeded in Rapid Run mode for 250 cycles in one lane on an Illumina Hi-Seq 2500.

### Sequence assembly, duplicate purging, and scaffolding

**Figure 2.**
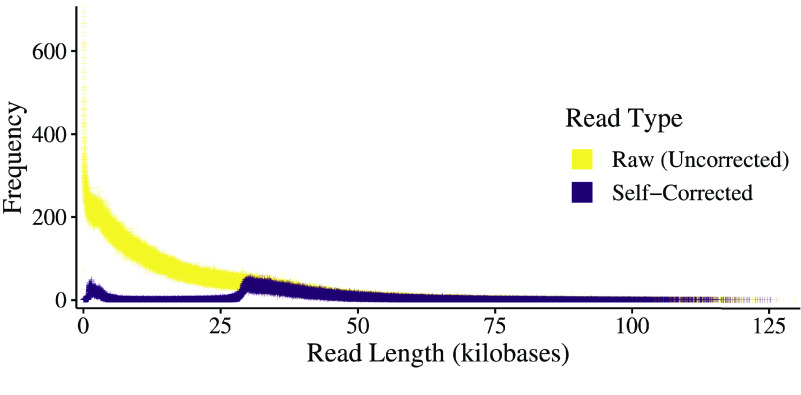
Frequency of Pacific Biosciences Read Lengths. The read length distributions before and after correction. The dramatic shift from raw to corrected reads is evident. Reads were corrected by consensus using the correction phase of Canu v1.8.

The PacBio CLR reads were self-corrected (Figure 2) and assembled with Canu v1.8 (RRID:SCR_015880) [18]. To get a haploid representation of the genome, duplicates were purged with purge_dups v1.2.5 (RRID:SCR_021173) [19]. The primary set of 329 contigs was selected for scaffolding with Omni-C data, which required reads to be mapped to the assembly before determining how to order and orient the contigs. The Omni-C reads were aligned following the Arima Genomics (San Diego, California, USA; https://arimagenomics.com) Mapping Pipeline commit #2e74ea4 (https://github.com/ArimaGenomics/mapping_pipeline), which relied on BWA-MEM2 v2.1 (RRID:SCR_022192) [20, 21], Picard v2.19.2 (RRID:SCR_006525) [22], and SAMtools v1.9 (RRID:SCR_002105) [23]. BEDTools v2.28.0 (RRID:SCR_006646) [24] was used to prepare the Omni-C alignments for scaffolding with SALSA commit #974589f (RRID:SCR_022013) [25, 26]. Before the scaffolding step, SALSA cleaned the assembly by breaking the misassemblies determined by the Omni-C read mappings. This set of contigs was then used simultaneously both for the remainder of the SALSA pipeline and for scaffolding with Rascaf v1.0.2 commit #690f618 (RRID:SCR_022014) [27] using the RNA-seq data from all the tissues aligned using HiSat v0.1.6-beta [28]. The two sets of scaffolds were combined using custom Python (https://www.python.org) scripts, which used the Omni-C scaffolds as starting points and added compatible joins from the RNA-seq evidence. Contaminations were removed from the final set of scaffolds as identified during the NCBI submission process; also, all gaps were adjusted to a fixed size (100 Ns). The repeat characterization was performed with RepeatMasker v4.1.2-p1 (RRID:SCR_012954) [28] – relying on RMBlast v2.11.0 (RRID:SCR_022710) [29, 30], TRF v4.09.1 (RRID:SCR_022193) [31], and hmmer v3.3.2 (RRID:SCR_005305) [32] – using Dfam v3.3 (RRID:SCR_021168) [33] and the RepBase RepeatMasker Library v20181026 (RRID:SCR_021169) [34, 35].

### Genome assembly validation

**Figure 3.**
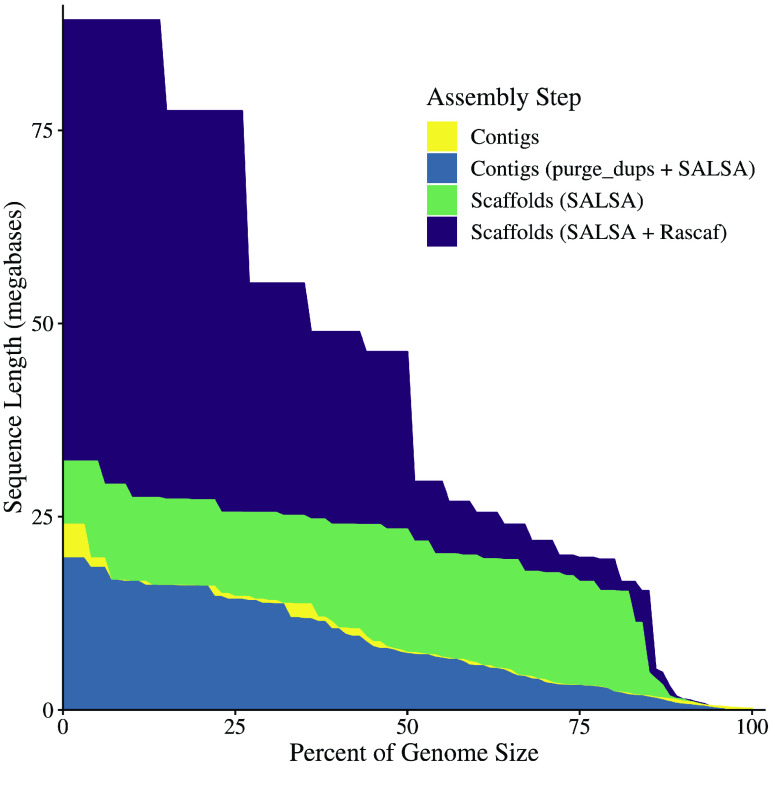
Area Under the NG curve for each Assembly Step. The NG curve and the area under it are plotted for the contigs and scaffolds. This visually demonstrates an increase in continuity from contigs to scaffolds. Scaffolding with RNA-seq data – which has minimal effect on its own (data not shown) – further increases the scaffold-level continuity. This plot also shows that duplicate purging and fixing misassemblies slightly reduced the contig-level continuity, as expected.

**Figure 4.**
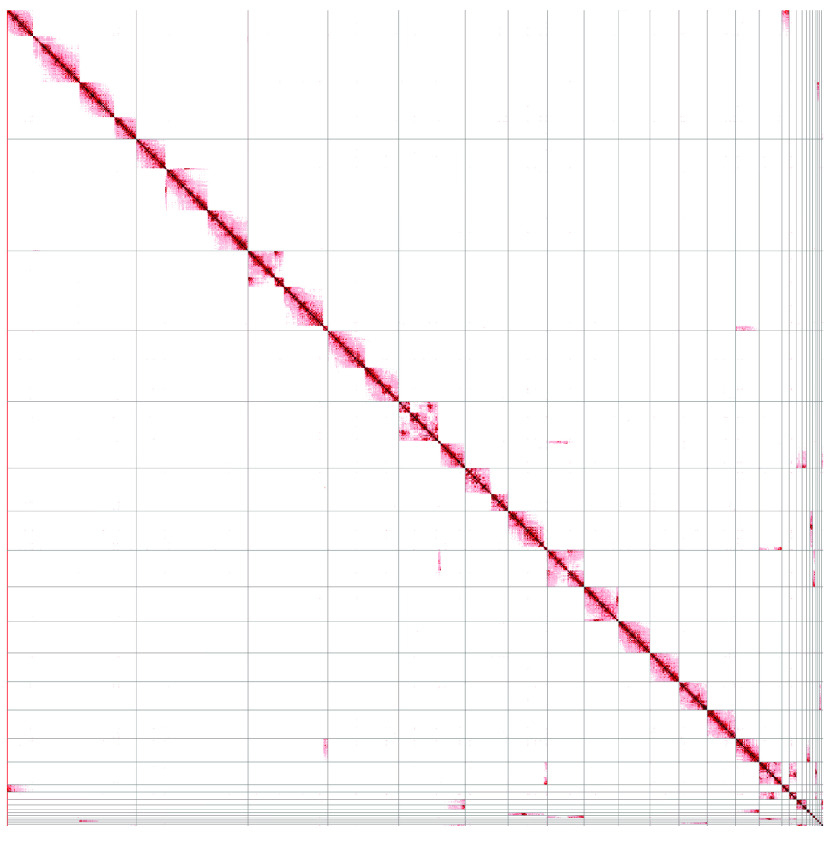
Hi-C Contact Matrix. In the context of scaffolding, Hi-C contact matrices show how correct the scaffolds are based on Hi-C alignment evidence. The longest 26 scaffolds are shown, ordered by descending length from top-left to bottom-right; the grey lines show the scaffold boundaries. Off-diagonal marks, especially dark and large ones, are possible evidence of mis-assembly and/or incorrect scaffolding. Regions with sharp edges similar to where the grey lines appear, but without the grey lines (e.g., three such locations occur in the top-left square), are joins between contigs in that scaffold that lack Hi-C evidence. The lack of Hi-C alignment evidence could suggest that these joins are invalid; however, evidence for these joins does exist from the RNA-seq alignments. The detection of any spurious joins would, at a minimum, require manual curation. Such curation would enable additional adjustments to fix the minor issues evidenced in the contact matrix.

At each phase of the assembly process, continuity statistics (e.g., N50 and auNG [36, 37]) were calculated with caln50 commit #3e1b2be (RRID:SCR_022015) (https://github.com/lh3/calN50) and a custom Python script (Figure 3; Table 3). The genome size (625.92 Mbp) provided to Canu and used for the computation of the assembly statistics was based on the C-value of 0.64 from Hardie and Hebert [14], as recorded in the Animal Genome Size Database [15]. The assembly completeness was also assessed at each phase using single-copy orthologs from the Actinopterygii set of OrthoDB v10 (RRID:SCR_011980) [38], as identified by BUSCO v5.3.2 (RRID:SCR_015008) [39, 40] (Table 4). The scaffolds were visually inspected using a Hi-C contact matrix (Figure 4) created with PretextView v0.1.4 (https://github.com/wtsi-hpag/PretextView) (RRID:SCR_022024) and PretextMap v0.1.4 (https://github.com/wtsi-hpag/PretextMap) (RRID:SCR_022023) with SAMtools v1.10 [23].

 The visual comparisons with other carangoid genomes were created for the cursory comparative genomics analysis and coarse validation via the observation of general similarities. Dot plots were generated using Mashmap v2.0 commit #ffeef48 (RRID:SCR_022194) [41] (-f ’one-to-one’ –pi 95 -s 10000) and the comparison of single-copy orthologs was created using ChrOrthLink commit #d29b10b (RRID:SCR_022195) after the assessment with BUSCO v3.0.6 [39] using the Vertebrata set from OrthoDB v9 [42]. The genome assemblies obtained from NCBI for these analyses were the following (alphabetical order): *Caranx melampygus* (bluefin trevally) [11], *Echeneis naucrates* (live suckershark) [43, 44], *Seriola dumerili* (greater amberjack) [43, 44], *Seriola quinqueradiata* (yellowtail) [45, 46], *Seriola rivoliana* (longfin yellowtail) [47], *Trachinotus ovatus* (golden pompano) [48, 49], and *Trachurus trachurus* (Atlantic horse mackerel) [50–53].

## Data validation and quality control

### Sequencing

CLR sequencing (PacBio) generated 3.74 M reads with a total of 58.25 Gbp, which is approximately 93× physical coverage of the genome. The mean and N50 read lengths were 15,591.278 and 27,441 bp, respectively. The longest read was 129,643 bp. The read length distribution is plotted in Figure 2. A summary of the results for the sequencing run is available in Table 1. This genome is the second for the *Caranx* genus and ranks highly in terms of N50 among the available carangoid genomes [49, 51]. 

**Table 1:** Sequencing information.

**Company**	Illumina	Illumina	PacBio
**Instrument**	Hi-Seq 2500	Hi-Seq 2500	Sequel I
**Mode**	High Output	Rapid Run	NA
**Sequencing type**	PE	Omni-C, PE	SMRT, CLR
**Duration**	125 cycles	250 cycles	10 h
**Specimen**	1	1	1
**Tissues**	Brain, Eye, Fin, Gill, Heart, Kidney, Liver, Muscle	Blood	Heart
**Molecule**	RNA	DNA	DNA
**Millions of read (Pair)s**	435.99	169.11	3.74
**Mean read length (bp)**	124.21	239.26	15,591.28
**Read N50 (bp)**	125	250	27,441
**Nucleotides (Gbp)**	108.30	80.92	58.25

The results from each type of DNA and RNA sequencing from *Caranx ignobilis*. PE  = Paired-end reads; SMRT  = single-molecule real-time sequencing; CLR  = continuous long-reads.

The RNA-seq from the eight tissues (i.e., brain, eye, fin, gill, heart, kidney, liver, and muscle) generated 435.99 M pairs of reads totaling 108.30 Gbp. Across all eight tissues, the mean and N50 read lengths were 124.21 and 125 bp, respectively. The combined results from all eight tissues are provided in Table 1, while the results from each tissue are available in Table 2. Omni-C sequencing generated 80.92 Gbp of data across 169.1 M read pairs. The N50 and mean read length were respectively 250 and 239.3 bp. The Omni-C results are also provided in Table 1 with the PacBio and RNA-seq data. The RNA-seq and Omni-C reads were not corrected, but the quality was assessed using fastqc [54].

**Table 2:** RNA sequencing details per tissue.

	Millions of read pairs	Mean read length	Read N50	Nucleotides (Gbp)
Brain	45.59	124.17	125	11.32
Eye	52.02	124.26	125	12.93
Fin	50.13	124.16	125	12.45
Gill	55.56	124.22	125	13.80
Heart	57.87	124.29	125	14.39
Kidney	58.73	124.16	125	14.58
Liver	58.25	124.23	125	14.47
Muscle	57.84	124.16	125	14.36
**All**	**435.99**	**124.21**	**125**	**108.30**

Results of the RNA sequencing of each tissue from one *Caranx ignobilis* individual. The eight tissues were spread across two lanes and run on an Illumina Hi-Seq 2500 in Rapid Run mode for 250 cycles to generate paired-end reads. Unless otherwise specified, lengths of nucleotide sequences are measured in base pairs (bp).

### PacBio CLR error correction

The correction process reduced the number of reads from 3.74 M to 656 K and the total number of bases from 58.3 Gbp to 23.9 Gbp, for an approximate physical coverage of 38.3×. The mean and N50 read lengths changed from 15,591 and 27,441 bp to 36,475 and 40,065 bp, respectively. The longest read was 126,321 bases. The distribution of the corrected read lengths can be viewed together with the raw read lengths in Figure 2.

### Genome assembly, duplicate purging, and scaffolding

The initial assembly generated by Canu comprised 1.8 K contigs for a total assembly size of 758 Mbp. That was a diploid assembly: both haplotypes were present and intermixed, separated whenever a bubble in the assembly graph prevented a single, reasonable contig. The duplicate purging to get a haploid representation of the genome (albeit with inevitable haplotype switching) and fixing misassemblies using evidence from Hi-C data yielded 343 contigs with a total assembly size of 605 Mbp. The mean contig length, N50, NG50 [55], and maximum contig length were 1.8 Mbp, 7.7 Mbp, 7.3 Mbp, and 19.6 Mbp, respectively. The L50 was 23, and the LG50 was 25. The area under the NG-curve (auNG) was 8.55 M. These values show modest reductions from the original Canu assembly (as expected), and they can be visualized through the auNG as shown in Figure 3 (also see Table 3).

**Table 3:** Continuity statistics.

	Contigs	Contigs (purge_dups)	Contigs (purge_dups + SALSA)	Scaffolds (SALSA)	Scaffolds (SALSA + Rascaf)	Scaffolds
**Sequences**	1,804	329	343	240	209	203
**Known bases**	757.523 Mbp	605.140 Mbp	605.140 Mbp	605.140 Mbp	605.140 Mbp	605.115
**Mean length**	0.420 Mbp	1.839 Mbp	1.764 Mbp	2.521 Mbp	2.895 Mbp	2.981 Mbp
**Max. length**	23.990 Mbp	23.990 Mbp	19.607 Mbp	32.157 Mbp	89.251 Mbp	89.251 Mbp
**NG50**	7.412 Mbp	7.412 Mbp	7.261 Mbp	23.385 Mbp	46.318 Mbp	46.303 Mbp
**NG90**	1.097 Mbp	0.950 Mbp	0.700 Mbp	1.386 Mbp	1.410 Mbp	1.410 Mbp
**LG50**	24	24	25	12	5	5
**LG90**	103	105	114	39	25	25
**auNG**	9.090 M	9.051 M	8.549 M	19.716 M	42.606 M	42.600 M
**Sequences with gaps**	-	-	-	40	35	35
**Gaps**	-	-	-	103	134	133
**Unknown bases**	-	-	-	51,500	52,027	13,300
**Mean Gap length**	-	-	-	500	388.261	100

Continuity statistics for the *Caranx ignobilis* genome assembly at the contig and scaffold level. Note that the auNG value is the area under the NG curve, not the N curve. The final set of scaffolds (far right column) is the same as “Scaffolds (SALSA + Rascaf” except that the identified contaminants were manually removed from the assembly and the gaps were unified to 100 Ns. Unless otherwise specified, all nucleotide sequences are measured in base pairs (bp).

 Paired-end Illumina reads, such as those produced from Hi-C or RNA-seq libraries, can provide information to order and orient contigs into scaffolds. However, they contain insufficient information for gap-filling procedures. Accordingly, the result of the assembly statistics should increase lengths, decrease the number of sequences, and leave the number of known bases unchanged. This pattern was evident in the assembly statistics from our iterative scaffolding procedure (Table 3). It is important to note that SALSA and Rascaf introduce gaps of unknown size, using fixed runs of 500 and 17 Ns, respectively, to represent such gaps. For submission to NCBI, these gaps were converted to a fixed length of 100 Ns; the evidence for whether the joins were supported by Hi-C or RNA-seq data was submitted in an accompanying file in AGP format (https://www.ncbi.nlm.nih.gov/assembly/agp/AGP_Specification). The NCBI submission process also identified minor contaminants in some sequences, which were manually removed. The final set of scaffolds had an NG50 of 46.3 Mbp and an auNG of 42.6 M (Figure 3; Table 3). All joins were represented in a contact matrix (Figure 4), showing the Hi-C evidence for the assembly. Some joins were poorly supported by the Hi-C evidence, which was not surprising as some joins were based on RNA-seq evidence instead. Without manual curation, it is difficult to ascertain whether any individual join is spurious.

The assembly completeness, as assessed with single-copy orthologs, was also evaluated at the contig and scaffold level (Table 4). The results suggest that the modifications made to the primary contig assembly from scaffolding did not significantly impact the complete assembly of single-copy orthologs. The final set of scaffolds had 3,545 complete single-copy orthologs (97.4% of 3,640 from the OrthoDB10 Actinopterygii set). Of these, 98.4% (3,488) were present in the assembly only once, and 1.6% (57) were present more than once. Fifteen (0.4%) and 80 (2.3%) single-copy orthologs were fragmented and missing from the assembly, respectively. Approximately 16.7% of the genome was comprised of repetitive elements (Table 5), similar to other Carangoid genomes: 16.9% for *Caranx melampygus* [11], 12.8% for *Pseudocaranx georgianus* [56], and 20.3% for *Trachinotus ovatus* [49].

**Table 4:** Summary BUSCO results.

	Contigs	Contigs (purge_dups)	Contigs (purge_dups + SALSA)	Scaffolds (SALSA)	Scaffolds (SALSA + Rascaf)	Scaffolds
**Complete**	97.6	97.5	97.5	97.4	97.5	97.4
Single Copy	85.8	96.0	96.0	95.9	95.9	95.8
Duplicated	11.8	1.5	1.5	1.5	1.6	1.6
**Fragmented**	0.3	0.5	0.5	0.5	0.4	0.4
**Missing**	2.1	2.0	2.0	2.1	2.1	2.2

Summary BUSCO results for the *Caranx ignobilis* genome assembly at the various contig and scaffold stages. Each value is the percentage of the single-copy orthologs (*n* = 3,640) in the Actinopterygii lineage dataset from OrthoDB v10.

**Table 5:** Summary of repeats.

	Copies	Length (Mbp)	Percent (%) of sequence
***Interspersed repeats***	**512,100**	**74.5**	**12.3**
SINE:	14,087	1.6	0.3
Penelope	5,290	1.0	0.2
LINE	62,098	12.5	2.1
LTR	15,550	3.6	0.6
DNA Transposon	237,928	33.1	5.5
Unclassified	177,147	23.6	3.9
***Tandem repeats***	**475,796**	**19.2**	**3.2**
Satellite	1,163	0.2	0.0
SSR	430,819	16.7	2.8
Low Complexity	43,814	2.3	0.4
**Rolling-circles**	**33,931**	**6.6**	**1.1**
**Small RNA**	**7,561**	**1.0**	**0.2**
**Total**	**1,029,388**	**100.8**	**16.7**

Summary of repeat content in the *Caranx ignobilis* genome assembly as reported by RepeatMasker [29] using the Dfam v3.3 [33] and RepBase RepeatMasker v20181026 [34, 35] repeat libraries.

### Comparison between the genomes of the giant trevally and other carangoids

**Figure 5.**
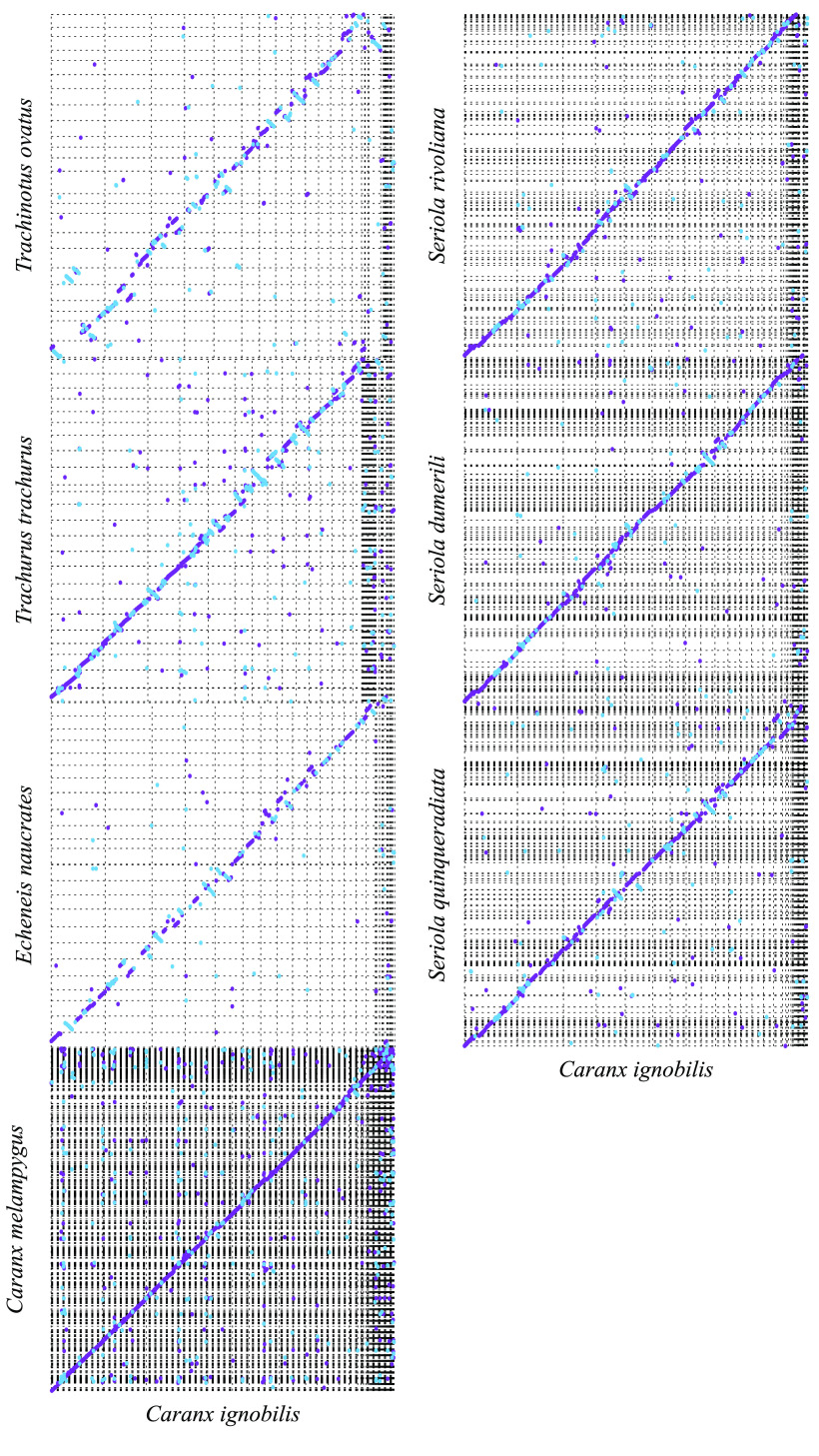
Dot Plot Comparisons with other Carangiformes (Carangoidei) Genomes. The dot plots show the relative continuity of the various segments of two genomes. The purple dots show segments that align in the positive orientation, blue in the negative. The *x*-axis is the *Caranx ignobilis* genome; the *y*-axes of each plot are the genomes of other carangoids. Dots off the diagonal indicate the structural variation between the genome assemblies. For assemblies that did not have duplicates purged to reduce the assembly to pseudohaplotypes (*Caranx melampygus* and *Seriola* spp.), the extra dots are presumably due to the alignment to the secondary copy.

**Figure 6.**
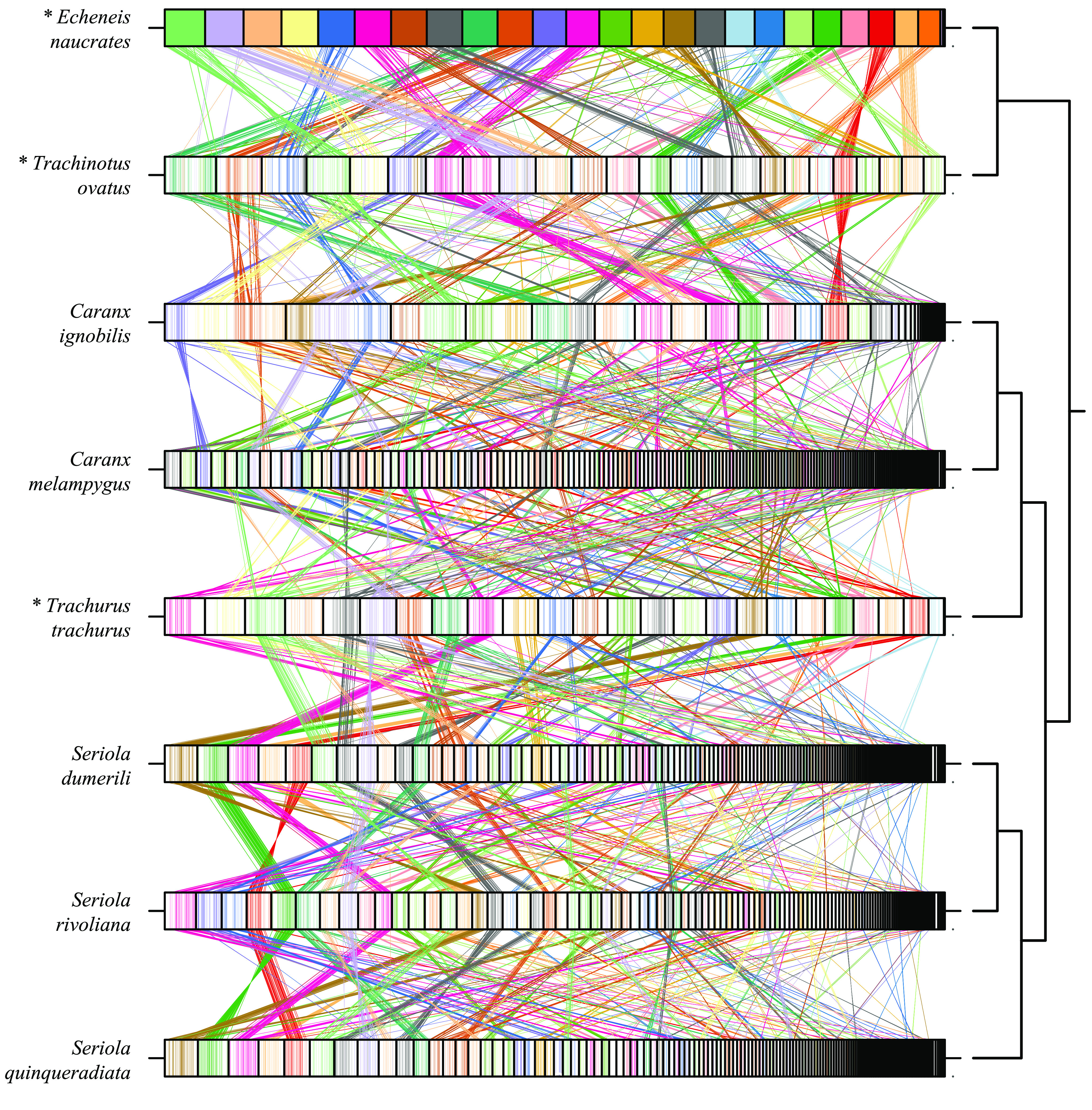
Single-copy Ortholog Comparisons with other Carangiformes (Carangoidei) Fishes. Single-copy orthologs from the Vertebrata set of OrthoDB v9 were identified with BUSCO v3.0.6 and visualized using ChrOrthLink. “Chromosomes” (usually contigs or scaffolds) are ordered based on length. Note that the sizes of the “chromosomes” are only relative to the other “chromosomes” in the same genome and cannot be compared between genomes. Chromosome-scale assemblies are marked with an asterisk. Colors are assigned based on the *E. naucrates* chromosomes, and individual lines are drawn tracking the placement of individual single-copy orthologs through each genome. Provided there are no structural rearrangements between different species’ genomes and the genomes are all of reliable quality, large blocks of colored lines should consistently appear together on single chromosomes across the various genomes. Sections of color appearing in blocks on more than one chromosome indicate regions where either chromosome rearrangements occurred or where there were scaffolding errors.

We compared the *C. ignobilis* genome with the published genomes of other carangoids spanning the carangoid phylogeny, including the live sharksucker (*Echeneis naucrates*) [43, 44], the golden pompano (*Trachinotus ovatus*) [48, 49], the yellowtail (*Seriola quinqueradiata*) [45, 46], the longfin yellowtail (*Seriola rivoliana*) [47], the greater amberjack (*Seriola dumerili*) [57, 58], the Atlantic horse mackerel (*Trachurus trachurus*) [50–52], and the closely-related bluefin trevally (*Caranx melampygus*) [11]. We generated dot plots to visualize the genome alignments and look for general similarities between the genomes (Figure 5). Some structural variations can be seen, but overall there do not appear to be regions of significant variation (e.g., inversions or frameshifts) between *C. ignobilis* and other carangoid species. We similarly compared the same genomes by visualizing the grouping of single-copy orthologs plotted along the assemblies (Figure 6). Large groupings of orthologs consistently appear between genomes, suggesting orthology not just between genes but also between larger genomic regions. However, at this scale and by comparing several genomes at once, it is difficult to make more refined inferences on the evolution of specific orthologs within Carangoidei. Additional information could be gleaned if all genomes were assembled at the chromosome scale and the sequences were ordered based on similarity.

 Specific patterns become difficult to inspect at the genome scale when the contigs and scaffolds are small. We observed that the longest scaffolds in the *C. ignobilis* assembly have many single-copy orthologs for more than one chromosome from chromosome-scale assemblies like *E. naucrates*. This observation suggests that an investigation of the validity of some of the *C. ignobilis* scaffolding joins should be performed before inferences are drawn about those regions. The joins based on Hi-C evidence are reasonably trustworthy. However, some joins based on RNA-seq data can be spurious under certain conditions — such as when RNA-seq reads split across introns and the mapping software mistakenly assigns each end to different genes with similar sequences (e.g., from duplication events or gene families). The true structure of the genome can be further elucidated by karyotype analysis, additional sequencing data (e.g., Ultra-long Nanopore (Oxford, England, UK)), and one-on-one comparisons with high-quality, chromosome-scale assemblies from related species. Ultimately, this genomic dataset is useful for future comparative studies on genome structure and evolution within Carangiformes and, more broadly, marine teleosts.

## Data Availability

Raw reads have been deposited in the National Center for Biotechnology Information (NCBI) Sequence Read Archive (SRA) [59–68] under BioProject PRJNA670456 [69] and BioSamples SAMN16516519–SAMN16516526﻿ and SAMN16629462 [70–78]. The genome assembly is associated with the same BioProject under the “container” BioSample SAMN18021194 [79] and can be found in GenBank under the accession JAFHLA000000000. See Table 6 for a complete list of the datasets and their mapping to BioSamples. The contigs, the scaffolds resulting from Hi-C evidence, and the scaffolds resulting from Hi-C or RNA-seq evidence are also available from the Center for Open Science’s (https://www.cos.io) Open Science Framework [80]. Snapshots of the code and other results files are available in the GigaDB repository [16]. Database information for raw sequences. All samples were collected from the same *Caranx ignobilis* specimen in April 2019 off the coast of O‘ahu (near Kaneohe, Hawai‘i, USA). They are combined under the BioProject PRJNA670456. The genome assembly is deposited in GenBank under accession JAFHLA000000000 with the “container” BioSample SAMN18021194.

## Availability of supporting source code and requirements

No significant computer programs were generated in this work. The commands used to generate these data are available as a shell script via the archived Bioinformatics Methods file in GigaDB [16]. Custom scripts referenced therein are available via GitHub.

- Project name: Caranx-ignobilis assembly-paper misc-scripts
- Project home page: https://github.com/pickettbd/caranx-ignobilis_assembly-paper_misc-scripts
- Operating system(s): Platform independent
- Programming language: Python
- License: MIT.

## List of abbreviations

auNG: area under the NG-curve; BYU: Brigham Young University; CLR: continuous long-read; DNASC: DNA Sequencing Center; NCBI: National Center for Biotechnology Information; SMRT: single-molecule real-time; SRA: Sequence Read Archive.
