# Supplementary material for: The genome of a giant (trevally): Caranx ignobilis
Source: GigaByte. 2022 Aug 30;2022:gigabyte67. doi: 10.46471/gigabyte.67 (PMC9694125; doi:10.46471/gigabyte.67)
Supplement: Peer review history

# Reviewer report: Review MS


Reviewer name and names of any other individual's who aided in reviewer Alison GouldDo you understand and agree to our policy of having open and named reviews, and having your review included with the published papers. (If no, please inform the editor that you cannot review this manuscript.)YesIs the language of sufficient quality?YesPlease add additional comments on language quality to clarify if needed
Are all data available and do they match the descriptions in the paper? YesAdditional CommentsAre the data and metadata consistent with relevant minimum information or reporting standards? See GigaDB checklists for examples &lt;a href="http://gigadb.org/site/guide" target="_blank"&gt;http://gigadb.org/site/guide&lt;/a&gt;YesAdditional CommentsIs the data acquisition clear, complete and methodologically sound?YesAdditional CommentsIs there sufficient detail in the methods and data-processing steps to allow reproduction?YesAdditional CommentsIs there sufficient data validation and statistical analyses of data quality? Not my area of expertiseAdditional CommentsIs the validation suitable for this type of data?YesAdditional CommentsIs there sufficient information for others to reuse this dataset or integrate it with other data?YesAdditional CommentsThis was a very clear and well-written manuscript presenting a whole genome assembly for the giant trevally. This will serve as an important resource for future researcher interested in this and other closely related species of fish. I. only have a few minor suggestions but overall, found the paper to be of high-quality. 
-It would be helpful to include the estimated genome size and BUSCO score in the abstract
-Include the species name on the X-axes of each column in Fig 5.
-Several of the tables (Table2, Table6, for example) don't seem necessary in the main text as they are not really discussed in the paper and could included as supporting material.Any Additional Overall Comments to the AuthorRecommendationAccept


# Reviewer report: Review MS


Reviewer name and names of any other individual's who aided in reviewer Song YueDo you understand and agree to our policy of having open and named reviews, and having your review included with the published papers. (If no, please inform the editor that you cannot review this manuscript.)YesIs the language of sufficient quality?YesPlease add additional comments on language quality to clarify if needed
Are all data available and do they match the descriptions in the paper? YesAdditional CommentsThe description of the data in the article is generally correct, but there are some inconsistencies. e.g. in line 186, the author used single-copy orthologs from the actinopterygii set of OrthoDB (v10) to assess assembly completeness but using vertebrate set for the comparison with other fish genomes. All the other species are also fish genomes, so why not use the same database (e.g. actinopterygii)?Are the data and metadata consistent with relevant minimum information or reporting standards? See GigaDB checklists for examples &lt;a href="http://gigadb.org/site/guide" target="_blank"&gt;http://gigadb.org/site/guide&lt;/a&gt;YesAdditional Commentsit is best to provide relevant information about protein-coding genes i thinkIs the data acquisition clear, complete and methodologically sound?YesAdditional CommentsIs there sufficient detail in the methods and data-processing steps to allow reproduction?NoAdditional Comments(1) It is better to provide detailed software parameters and the description of how to assemble contigs into scaffold is not clear enough.
(2) The method of how to identify single-copy orthologs is not clearly describedIs there sufficient data validation and statistical analyses of data quality? NoAdditional CommentsI don't think it's enough to just rely on single-copy orthologs and/or synteny blocks to assess the genome quality, maybe it would be better to add some others, e.g. reads mapping?Is the validation suitable for this type of data?YesAdditional CommentsIs there sufficient information for others to reuse this dataset or integrate it with other data?YesAdditional CommentsAny Additional Overall Comments to the Author(1) In line 223, the authors just provide how many scaffolds there are in the final assembly version, but how many chromosomes are assembled and how about the proportion of scaffold or contigs which have been located into the chromosomes. These information is not found in the MS. Note that there is already published a genome for this genus in NCBI, but only at the contig level, if using the Hi-C data could provide the chromosomal level one, I think it would be more useful

(2) In line 252, I noticed there was not mentioned about gene sets, especially the protein-coding genes, how many coding genes are there in this genome?

(3) In figure 4, many cross-linking intensities are not obvious, which may be related to sequencing depth of Hi-C data, I can't figure out how many chromosomes are there in the final assembly from this diagram.

(4) Some minor bugs, in FIGURE CAPTIONS, figure 6, here the author used the ray-finned fish, right? I think it is a mistake here, cause in line 186, the author mentioned vertebrata set. RecommendationMinor Revision


# Reviewer report: Re-Review MS


Comments on revised manuscriptThe author has responded to the corresponding questions, recommended accepting the manuscript


# Editor report: Accept


Comments to the AuthorPlease update the DOI in figure 1.
